# Analysis of static postural balance using a 3d electromagnetic system

**DOI:** 10.1590/S1808-86942010000600018

**Published:** 2015-10-19

**Authors:** José Ailton Oliveira Carneiro, Taiza Elaine Grespan Santos-Pontelli, José Fernando Colafêmina, Antonio Adilton Oliveira Carneiro, Eduardo Ferriolli

**Affiliations:** 1 Master's degree, doctoral student - Department of Internal Medicine, Ribeirão Preto Medical School, São Paulo University - FMRP-USP; 2 Doctoral degree, Department of Neuroscience and Behavioral Science, Ribeirão Preto Medical School, São Paulo University - FMRP-USP; 3 Doctoral degree, faculty member of the Department of Ophthalmology, Otorhinolaryngology and Head & Neck Surgery, Ribeirão Preto Medical School, São Paulo University - FMRP-USP; 4 Post-doctoral degree, faculty member of the Physics and Mathematics Department of the School of Philosophy, Sciences and Letters, Ribeirão Preto da Universidade de São Paulo, FFCLRP-USP; 5 Post-doctoral degree, faculty member, Department of Internal Medicine, Ribeirão Preto Medical School, São Paulo University - FMRP-USP. Ribeirão Preto Medical School, São Paulo University - FMRP-USP (Faculdade de Medicina de Ribeirão Preto/FMRP-USP)

**Keywords:** young adult, postural balance, sensory deprivation

## Abstract

**Abstract:**

Early detection of postural disorders is essential for timely interventions in patients with imbalance.

**Aim:**

A pilot study describing a new tool for evaluating static postural balance.

**Study design:**

A cross-sectional study of a contemporary series.

**Material and Method:**

Twenty-five volunteers (15 women and 10 men) were evaluated. The mean age was 25.8 ± 4.2 years, the mean weight was 63.9 ± 13.1Kg, the mean height was 1.68 ± 0.08 m and the body mass index was 22.3±3.3kg/m2. Posturography was done by analysing postural sway with an electromagnetic system; a sensor was attached to the skin over the spinous process of the first thoracic vertebra. Tests were carried out with the subjects in the orthostatic position for 90 seconds, with eyes opened(EO) and closed(EC) on stable and unstable surfaces.

**Results:**

When the influence of the surface was analyzed (stable x unstable) in the EO condition, there were significant differences in the middle-lateral parameters (m-l) (p=0.004) and total path (p=0.01), and in the m-l (p=0.004) and total (p=0.014) speed. In the EC condition, there were significant differences in all parameters (p<0.001). The influence of the vision was observed in all parameters only on unstable surfaces (p<0.05).

**Conclusion:**

The new tool was efficient for analysing postural sway.

## INTRODUCTION

Evaluating balance in medical practice is essential for the early detection of postural disorders and for appropriate interventions in patients with balance disorders; this provides a better understanding and recognition of differences among individuals.

When individuals are in the upright position, their bodies move as a simple inverted pendulum; muscles that cross the main rotation axis - the ankle - are able to control the position of the body center of mass (CM).^1^ Other postural synergies have been amply characterized as automatic responses to external perturbation because of unexpected forces on support surfaces^2^, ^3^ or anticipatory adjustments preceding voluntary movements.^4^, ^5^, ^6^, ^7^

The ability to maintain posture depends on sensory information, which is necessary for the nervous system to detect anticipatorily and suddenly any external perturbation, and to generate coordinated responses to bring back the body center of mass to the basis of support.^8^, ^9^ Thus, afferent information from the vestibular, visual, proprioceptive and interoceptive systems are essential for integrating the body with space and to maintain postural balance.^10^, ^11^, ^12^, ^13^, ^14^, ^15^

At present, there are several tools for quantifying body balance, such as force platforms, baropodometry, and 3D electromagnetic sensors. Using these tools, however, may be expensive and time-consuming, and requires expert labor to acquire and analyze data; thus, these tools are not used as often as wished in clinical and some research settings.

The Clinical Test of Sensory Interaction and Balance (CTSIB), which employs computerized dynamic posturography, was developed to identify the contribution of the three balance sensory systems (vision, vestibular, and somatosensory systems).^16^, ^17^, ^18^ This test separates theses sensory contributions by removing or distorting the surface or vision.^16^, ^19^, ^20^ The cost of equipment is one of the major difficulties in using computerized posturography in balance testing (force platform); the other is transporting it to other sites.

An available tool that has not been evaluated adequately for investigating postural balance is the 3D electromagnetic sensor system; its advantages are lower cost and ease of transportation to different sites. At present there are several models of this device, each with a different number of sensors. These may be employed in multisegmental posturography because minor oscillations of different body segments - and thus a direct investigation of posture controlling kinematics - can be studied.^7^, ^12^, ^21^

Accornero et al. used this technology with two sensors (one on the head and the other on the lumbar area) to study volunteers on a stable surface with eyes open and closed. Head sensors, however, limit the possibility of analyzing several degree of freedom of this bodily segment.

The purpose of this study was to evaluate a new method for analyzing static postural balance by employing a 3D electromagnetic system with a single sensor, during a modified clinical test of sensory interaction and balance (mCTSIB). The advantages of this method are its portability and reasonable cost; it also yields simple and easily interpreted data.

## MATERIALS AND METHODS

This was a contemporary cohort cross-sectional study that enrolled 25 healthy volunteers (15 female and 10 male) aged 18 to 35 years. Volunteers were chosen from students and staff of a public university campus. All volunteers were informed in detail about the methods of this study and signed a free informed consent form. The institutional review board approved the study (Process 244/2008).

A clinical history was taken of volunteers to identify possible medical conditions. Exclusion criteria were the presence of vestibular, neurologic, osteomuscular, cardiovascular, and psychiatric disorders, and visual difficulties without corrective lenses.

The weight and height of volunteers was measured to calculate the body mass index (BMI). The body weight was measured on Filizola digital scales (0.1 kg intervals) with the subjects using light clothes and no shoes. Body height was measured with a non-extendible 0.5 cm graded vertical bar stadiometer. All volunteers were classified as sedentary according to the International Physical Activity Questionnaire (IPAQ, short version 8.0).

**Figure 1:**
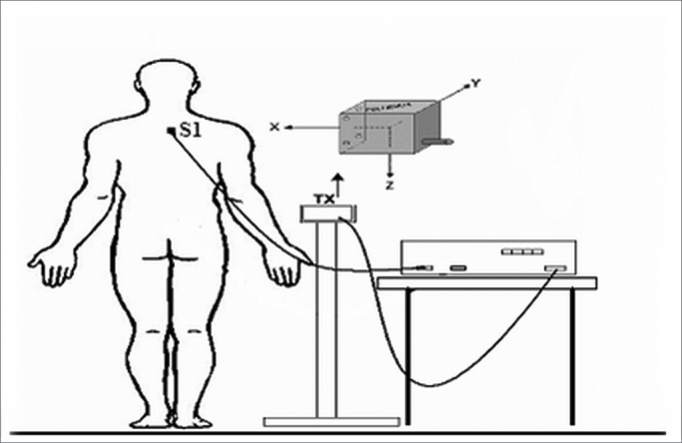
Location of electromagnetic sensors. Tx: transmitter and the 3 planes. S1: 1st thoracic vertebra.

A POLHEMUS® Patriot (Polhemus, EUA) electromagnetic sensor system was employed to measure the 3D position and spatial orientation of volunteers. The relative position (x, y, z coordinates and Euler's angles - θ, ϕ, ρ) of the receptor sensor fixed over the spinous process of the 1^st^ thoracic vertebra of each volunteer was measured. The system transmitter was placed over an uncoupled support from the volunteer's body at a 40 cm distance and at the height of the sensor (Fig. 1). Data were transferred to a laptop computer - 100 samples per second through an USB connection and a LabView 8.0 environment control and processing interface. Data processing took place simultaneously with digitizing so that the oscillation profiles of volunteers could be seen in real time in an independent graphic presentation from the three x,y,z coordinates of the recording. These coordinates are the anteroposterior, middle-lateral and cranio-caudal movements.

The surfaces were a wood platform (height - 1 cm, length - 50 cm, width - 50 cm) and a 30 kg/m^3^ density foam platform (height - 5 cm, length - 50 cm, width - 50 cm).^22^

Volunteers remained in orthostatism with arms held loose alongside the body and feet slightly apart over a reference surface during data acquisition. They were instructed to remain static, not moving the upper limbs, ankles and feet over the wood platform (stabile surface) and on the foam platform (unstable surface). The mCTSIB, which consists of four sensory condition, was applied in the following order: condition 01: stable surface, eyes open (OASE); condition 02: stable surface, eyes closed (OFSE); condition 03: unstable surface, eyes open (AOSI); condition 04: unstable surface, eyes closed (OFSI). In the eyes open conditions, subjects were asked to fix their gaze on a point 1.5 meters facing them. Each sensory condition was assessed during 90 seconds.

Data in the laptop were processed mathematically using software designed for this purpose in the LabView 8.0 environment and transformed into maximum displacement, velocity and path values.

The maximum anteroposterior displacement was the largest amplitude of anteroposterior movement (a-p); maximum middle-lateral displacement was the largest amplitude of middle-lateral movement (m-l). The path (total displacement) was defined as the total path taken by the body during the data acquisition time in the anteroposterior and middle-lateral directions. The mean velocity was calculated as the ratio between the path (total displacement) and time. The calculation variable/height was done for the corrected statistical data analysis according to the height of each volunteer.

The Shapiro-Wilk test was applied to test whether variables were distributed normally. Student's t test was applied to analyze the physical characteristics of the study population. The ANOVA test and Tukey's post hoc test were applied for comparisons among sensory conditions - bicaudal test at 5% significance. Pearson's correlation test was applied to analyze the correlation among variables without and with volunteer height correction. The SPSS (Statistical Package for the Social Sciences^®^) version 16.0 was used for electronic data processing; the Origin^®^, version 6.0 (Mi-crocal Origin^®^, 6.0, EUA) statistical software was used for building the charts.

## RESULTS

The mean age of the study population was 25.8±3.6 years, the mean height was 1.69±0.08 m, the mean weight was 64.4±2.5 Kg, and the mean BMI was 22.3±3.3 kg/m^2^.

Table 1 presents the values of variables in the open eyes and closed eyes conditions on stable and unstable surfaces without and with subject height correction. The analysis of the influence of vision on a stable surface revealed no significant differences in any of the study parameters compared to open eye and closed eye conditions. However, there were statistically significant differences in all parameters when these two conditions were compared on the unstable surface (p<0.05).

**Table 1:** Values of the variables: maximum displacement, path and velocity of volunteers in several sensory conditions without and with data correction by the height of each volunteer

VARIBLES	PRESENCE OF DATA CORRECTION BY HEIGHT	OASE	OFSE	OASI	OFSI
Max. displac. - a-p (cm) (mean±standard deviation)	without with	2.37±0.97 1.62±0.55	2.78±1.23 1.61±0,69	3.51±1.47 2.08±0.85	5.14±2.13 b d 3.03±1.18 b d
Max. displac. - m-l (cm) (mean±standard deviation)	without with	1.65±1.14 0.96±0.64	1.33±0.58 0.78±0.33	2.28±0.7 1.36±0.42	3.01±1.12 b d 1.78±0.63 b d
Path - a-p (cm) (mean±standard deviation)	without with	76.6±14.37 45.88±10.28	86.93±16.09 49.66±11.2	90.45±25.69 54.33±17.62	110.95±27.78 b d 66.31±18.58 b d
Path - m-l (cm) (mean±standard deviation)	without with	43.42±10.99 25.85±6.75	44.7±11.30 25.96±7	54.62±11.85 c 32.61±7.79 c	64.85±11.45 b d 38.46±6.08 b d
Total path (cm) (mean±standard deviation)	without with	118.46±19 70,82±13,71	128.42±21.16 72,67±14,92	142.67±32.9 c 85,55±23,1 c	168.68±34.37 b d 100,7±23 b d
Velocity - a-p (cm/s) (cm) (mean±standard deviation)	without with	0.85±0.15 0.51±0.11	0.97±0.17 0.55±0.12	1.00±0.28 0.60±0.19	1.23±0.31 b d 0.73±0.2 b d
Velocity - m-l (cm/s) (cm) (mean±standard deviation)	without with	0.48±0.12 0.28±0.07	0.5±0.12 0.28±0.07	0.60±0.13 c 0.36±0.08 c	0.72±0.12 b d 0.42±0.06 b d
Total velocity (cm/s) (cm) (mean±standard deviation)	without with	1.31±0.21 0.78±0.15	1.43±0.23 0.81±0.16	1.58±0.36 c 0.95±0.25 c	1.87±0.38 b d 1.12±0.25 b d

OASE: open eyes, stable surface; OFSE: closed eyes, stable surface; OASI: open eyes, unstable surface;

OFSI: closed eyes, unstable surface; a-p: anteroposterior; m-l: middle-lateral.

a = significant difference (p<0.05) between OASE X OFSE conditions - Influence of vision

b = significant difference (p<0.05) between OASI X OFSI conditions - Influence of vision

c = significant difference (p<0.05) between OASE X OASI conditions - Influence of surface

d = significant difference (p<0.05) between OFSE X OFSI conditions - Influence of surface

The analysis of the influence of surfaces (stable x unstable) for postural balance without correcting for height of individual in the open eye condition revealed significant differences in the parameters m-l path (p=0.004), total path (p=0.014), m-l velocity (p=0.004) and total velocity (p=0.014). There were significant differences in all parameters in the closed eye condition (p<0.001).

A strong correlation (r ≥ 0.95; p<0.001) was found among all variables without and with correction for individual height.

**Figure 2:**
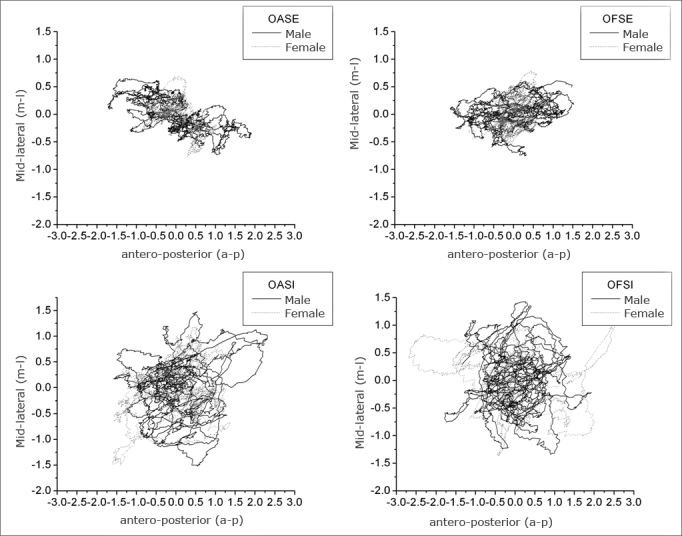
Statokynesigram of a man and woman in all anteroposterior (a-p) and middle-lateral (m-l) sensory conditions.

Figure 2 presents the statokinesigrams in different sensory conditions for a man and a woman with respective heights of 1.75 m and 1.59 m. These figures show the total postural oscillation path of an individual during 90 seconds, and represent postural oscillation maps in the a-p and m-l directions.^23^ These charts show that the area of oscillation increases as less sensory information is available. This analysis yields additional conclusions on postural balance of an individual during the acquisition time.

**Figure 3:**
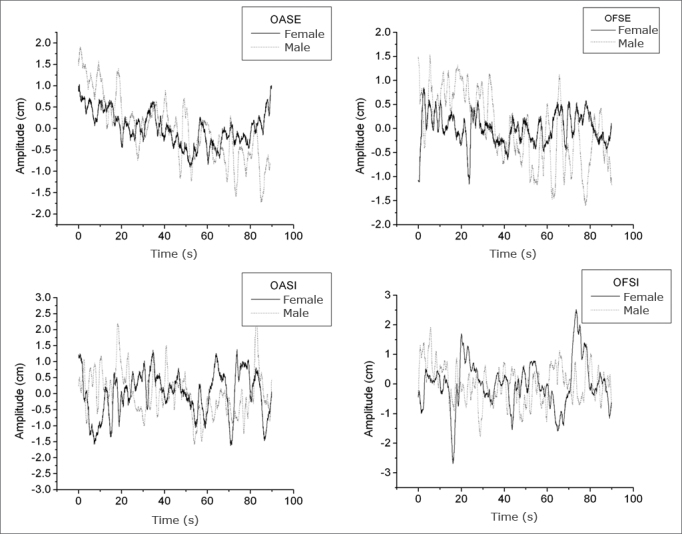
Stabilogram of a man and a woman in all anteroposterior (a-p) sensory conditions.

**Figure 4:**
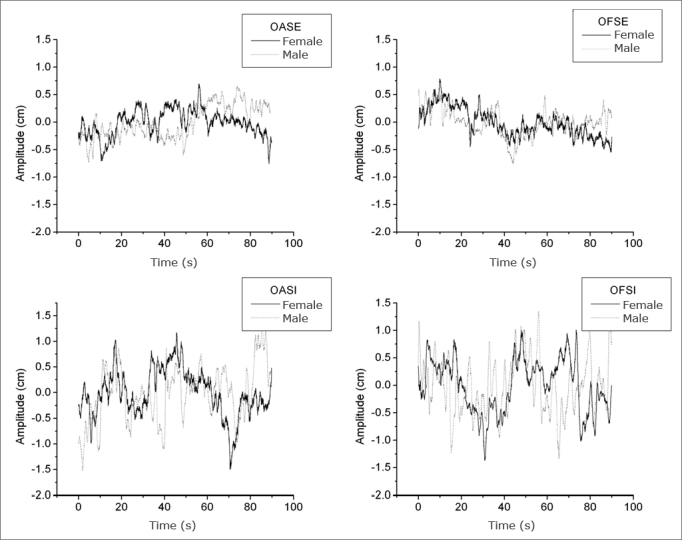
Stabilogram of a man and a woman in all middle-lateral (m-l) sensory conditions.

Figures 3 and 4 present the stabilograms at different sensory conditions for a man and a woman with respective heights of 1.75 m and 1.59 m. These figures show the postural oscillation amplitudes relative to acquisition time in the a-p and m-l directions, which yields a more detailed analysis of oscillation amplitudes at a specific moment.^23^ Stabilograms make it possible to identify test periods in which subjects attained maximum oscillation peaks (highest risk of falls). Figure 3 shows the postural oscillation amplitude in the a-p direction, and Figure 4 shows the same in the m-l direction.

## DISCUSSION

Using a 3D electromagnetic device (Polhemus^®^) with a single sensor placed over the thoracic spine was effective for recording information about postural oscillation and for detecting differences among sensory conditions. Our group developed specific software for acquiring, treating and analyzing information about human posture collected with this device. Therefore, the method became easy to apply and data interpretation and analysis was facilitated.

The CTSIB is traditionally used for evaluating postural balance; it consists of a combination of visual sensory conditions (eyes open, eyes closed, and visual conflict) and a surface (normal and imprecise orientation). The resulting six sensory conditions help identify which sensory information the patient primarily trusts in for spatial orientation and which sensory conflict situations cause instability.^24^ The mCTSIB was used in this study; according to Rosa et al. (2006),^25^ it yields global evidence of sensory function and body balance, but does not provide specific information about each system singly (visual, somatosensory and vestibular systems).

The 3D system and testing in different sensory conditions enabled us to analyze the influence of vision (eyes open and closed) and the surface (stable and unstable) for maintaining balance. As noted in several studies that applied the force platform,^26^, ^27^, ^28^, ^29^ the 3D system (Polhemus^®^) was also able to demonstrate significant differences among open and closed eyes on an unstable surface, which traditionally do not occur on a stable surface. This suggests that proprioceptive information is more relevant than visual information for static balance.

According to Chiari et al., height is a relevant parameter for analyzing balance in an inverted pendulum model.^30^ However, correcting data by the height of subjects showed that it had little effect on the postural balance of samples evaluated with the 3D electromagnetic device (Polhemus®). Kim et al. found similar results using force plataforms.^31^

The 3D electromagnetic sensor system (Polhemus^®^) has several uses; it is able to measure the real time spatial position and orientation of an object with a 2 mm accuracy. This device, however, is not often used for posturography. As far as we know, a single study by Accornero et al. (1997) has been published using this technology.

Based on our study, we propose a different method by employing a single sensor in a different position, which was able to measure postural balance parameters and detect changes in sensory conditions. We also developed a new data acquisition and analysis software. Furthermore, while Accornero et al. studied volunteers only on stable surfaces, our work evaluated postural oscillation on stable and unstable surfaces.

The limitations of this equipment are the data acquisition environment - places with considerable structural metal content or major electrical systems should be avoided, which may affect the magnetic field and interfere with data gathering. This was a pilot study enrolling only young healthy subjects. Additional studies are needed to define the sensitivity of this method so that parameter differences may be detected in several populations. Adding a stimulus that causes visual conflict to then proceed with the original CTSIB, and using a moving surface to evaluate the dynamics of postural control, may increase significantly the scope of this device.

## CONCLUSION

We present an affordable method employing an easily transported device for analyzing postural oscillation in different sensory conditions. Data on healthy subjects are presented to be later compared with other populations and individuals with several diseases. This tool may become useful for helping define appropriate rehabilitation measures and to provide information to be used when monitoring the results of any given therapy.
